# Association Study on Risk Factors for Major Infectious Diseases in Dogs and Cats in Shenzhen, China

**DOI:** 10.3390/ani16010049

**Published:** 2025-12-24

**Authors:** Yao Peng, Runchang Lin, Wanxing Xie, Rongjie Huang, Shunping Cai, Yinyi Liang, Qida Lin, Gen Li, Xiaofeng Guo, Bowen Lin, Jun Luo

**Affiliations:** 1 College of Veterinary Medicine, South China Agricultural University, Guangzhou 510642, China; pengyao2027@163.com (Y.P.); 19120393811@163.com (W.X.); rjhuang@stu.scau.edu.cn (R.H.); bobysmail@163.com (Y.L.); ligen223@126.com (G.L.); xfguo@scau.edu.cn (X.G.); 2 Shenzhen Inspection and Testing Center of Agricultural Product Quality and Safety, Shenzhen 518000, China; sz1322755037@163.com (R.L.); 13392885552@vip.189.cn (S.C.); fatcatlqd@163.com (Q.L.); 3 Zhaoqing Branch Center of Guangdong Laboratory for Lingnan Modern Agricultural Science and Technology, Zhaoqing 526000, China

**Keywords:** dog, cat, prevalence, infectious disease risk factors

## Abstract

This study investigated the prevalence of 11 common pathogens in dogs and cats in Shenzhen, China, from January 2022 to March 2024, aiming to enhance the understanding of their epidemiological characteristics for improved disease control strategies. Diagnostic testing for the target pathogens was performed using polymerase chain reaction (PCR), colloidal gold test strips, or fluorescence immunoassay. Statistical analysis revealed that among 13,134 cats, Feline Panleukopenia Virus (FPV) showed the highest prevalence (35.83%), followed by Feline Calicivirus (FCV, 26.20%), Feline Infectious Peritonitis Virus (FIPV, 22.00%), and Feline Herpesvirus (FHV, 15.76%). Among 3626 dogs, Canine Parvovirus (CPV) and Canine Distemper Virus (CDV) were predominant, showing a prevalence of 54.55% and 42.83%, respectively. Risk factor analysis showed that most infections occurred in unvaccinated animals and young individuals (<1 year old), with higher incidences in winter and spring. Logistic regression indicated that sex, age, and season were significantly associated with FPV, FHV, and FIPV infections, while age and season were associated with FCV, CPV, and CDV infections (sex showed no association). This study contributes to the epidemiological knowledge of common infectious diseases in dogs and cats, providing a theoretical basis for disease prevention in dogs and cats.

## Simple Summary

From January 2022 to March 2024, a survey was conducted in Shenzhen, China to investigate 11 common pathogens in 13,134 cats and 3626 dogs, aiming to optimize disease control by understanding their epidemiological features. Among these 11 pathogens, 7 are feline-specific, and 7 are canine-specific. Feline Panleukopenia Virus (35.83%) was the most prevalent in cats, while Canine Parvovirus (54.55%) and Canine Distemper Virus (42.83%) were more prevalent in dogs. Unvaccinated, under-1-year-old animals had higher infection risks, with increased incidence in winter and spring. Age and season correlated with most infections, and gender with some. The study provides new epidemiological insights for canine and feline infectious diseases, laying a theoretical basis for targeted prevention to protect pet health and reduce transmission impacts.

## 1. Introduction

Dogs and cats are important companion animals for human beings, and infectious diseases still seriously threaten their health. For cats, a variety of common pathogens can cause high morbidity and mortality. FPV (Feline Parvovirus) is a parvovirus in the family Parvoviridae and leads to high fever, severe vomiting, diarrhea, and leukopenia, with a mortality rate of 25–100% depending on severity [1,2]. FCV (Feline Calicivirus), an RNA virus of the Caliciviridae family, primarily spreads through oral and nasal secretions, and infection can result in clinical symptoms such as upper respiratory tract disease, gingivostomatitis, and limping syndrome [3]. The virus is widely distributed across various countries and cat populations, with a prevalence of 0% to 29% in healthy cats. The high genetic plasticity of FCV facilitates immune evasion and sustains long-term infections [4]. Feline Herpesvirus (FHV) causes viral rhinotracheitis with sneezing, fever, respiratory rales, and keratoconjunctivitis, with a 50% mortality rate and lifelong latent carriage [5,6,7]. Feline Infectious Peritonitis (FIP), caused by a variant of Feline Coronavirus (FCoV), is a fatal disease characterized by peritonitis, ascites accumulation, and eosinophilic granulomatous lesions of multiple organs [8]. It is the most common disease that causes cat death in recent years, with the mortality rate approaching 100% once symptoms appear. Cats carrying FCoV can transmit it to other cats in the same household via the fecal-oral or nasal route. When the coronavirus mutates, it can cause the fatal disease FIP [9]. The early symptoms of FIP are non-specific, and it is difficult to distinguish it from Feline Enteric Coronavirus (FECV), another pathological type caused by FCoV, using conventional diagnostic methods [10].

Canine Distemper Virus (CDV) and Canine Parvovirus (CPV) are high-incidence infectious diseases of dogs worldwide. CDV causes Canine Distemper, with clinical symptoms including biphasic fever, skin rash, respiratory signs, and potential neurological symptoms such as encephalitis, leading to sequelae or poor prognosis [11]. Liang et al. conducted a meta-analysis of relevant global articles published between 1983 and 2023 and found that the CDV positive rate was 22.6% among 8582 tested samples from minks, foxes, and raccoon dogs across 12 countries [12]. This result confirms the widespread prevalence of the virus in carnivores and its potential threats. CPV causes acute hemorrhagic enteritis, vomiting, and suppurative myocarditis [13]. Studies have shown that unvaccinated puppies infected with CPV face a mortality rate of up to 90%, and even with aggressive treatment, the mortality rate remains around 20–25% [14]. There is an increased risk of chronic gastrointestinal diseases in dogs cured after infection with CPV [15].CPV also poses a risk of cross-species transmission [16], and is often associated with co-infection with pathogens such as CDV [17].

The threat of companion animal infectious diseases to global public health and welfare is increasingly prominent, and regional heterogeneity in pathogen characteristics and epidemiological dynamics further exacerbates this risk, leaving gaps in targeted prevention and control systems. Although vaccines exist for these diseases, they cannot completely block transmission. Thus, analyzing risk factors for canine and feline infections is crucial. This study analyzed medical records from Shenzhen pet clinics to identify associations between sex, age, season, vaccination status, and disease occurrence, providing data for local disease prevention.

## 2. Materials and Methods

### 2.1. Case Collection

From January 2022 to March 2024, 16,760 cases (3626 dogs and 13,134 cats) were collected from Dachong, Shixia, Yuehai, Xixiang, Xinruipeng, and Ruipai Pet Hospitals in Shenzhen. Owner-reported animal information included age categories (<1 year, 1–8 years, ≥8 years), sex, vaccination status (yes/no), and sample collection season (winter: December-February; spring: March-May; summer: June-August; autumn: September-November). Vaccination status referred to immunization with Fel-O-Vax^®^ PCT (feline, inactivated vaccine, Zoetis Inc., Parsippany, NJ, USA) or VANGUARD^®^ PLUS 5/8 (canine, polyvalent vaccine, Zoetis Inc., Lincoln, NE, USA). Fel-O-Vax^®^ PCT is primarily designed to prevent FPV, FCV, and FHV. VANGUARD^®^ PLUS 5 is indicated for the prevention of CDV, infectious hepatitis caused by canine adenovirus type 1 (CAV-1), respiratory disease induced by canine adenovirus type 2 (CAV-2), Canine Parainfluenza Virus (CPIV), and CPV. VANGUARD^®^ PLUS 8 builds on the preventive coverage of VANGUARD^®^ PLUS 5, adding protection against canine coronavirus disease and leptospirosis caused by Leptospira canicola and Leptospira icterohaemorrhagiae. Unfortunately, due to the heterogeneous clinical record standards across different regions where the cases in this study were sourced, only partial cases had complete vaccination records. Case distribution is shown in Table 1 and Table 2.

### 2.2. Detection Methods

All samples in this study were sourced from the medical record databases of clinical institutions, with the inclusion criteria clearly defined as “cases presenting with typical clinical symptoms related to the target pathogens at the time of consultation; collecting whole blood, anal swabs, or ocular-nasal-pharyngeal swab for diagnostic testing including PCR, colloidal gold test strips, or fluorescent immunoassay, with confirmed positive results.” Although the study cases originated from different regions, leading to variations in detection methods, reagents, and instruments, each antigen was tested using one or more detection methods.

### 2.3. Sample Treatment

Ocular-nasal-pharyngeal swabs were collected for the diagnostic detection of FHV, FCV, CDV, and CAV; anal swabs for the diagnostic detection of FPV, FIPV, CPV, and CDV; and EDTA-anticoagulated whole blood for the diagnostic detection of TOXO, *Leptospirosis*, *Canine babesiosis*, and *Leishmania* spp. The tips of the swabs (ocular, nasal, pharyngeal, and anal) should be broken off and placed into sample preservation solution, followed by thorough vortexing for subsequent use. For whole blood samples, 200 μL of EDTA-anticoagulated whole blood was added to sample preservation solution, vortexed thoroughly, and stored for later use.

### 2.4. Polymerase Chain Reaction (PCR)

All 11 target pathogens were detected using the Real Time PCR Detection Kits (Shenzhen Gangzhu Medical Technology Co., Ltd., GZMEDTECH, Shenzhen, China). The protocol was as follows: Viral DNA/RNA was extracted from samples using a nucleic acid extraction kit on the GZ-NP2 Automatic Nucleic Acid Extractor (GZMEDTECH, Shenzhen, China). A 20 µL aliquot of the extracted nucleic acid was added to the PCR amplification reagent, followed by thorough dissolution and mixing. The reaction mixture was then subjected to amplification and detection using the GZ-8 Plus Portable Real-Time PCR Instrument (GZMEDTECH, Shenzhen, China), and a final detection report was generated.

### 2.5. Colloidal Gold Test Strips

Colloidal gold test strips (Luoyang Pu-tai Biological Technology Co., Ltd., PLKWT, Luoyang, China) were used for the diagnostic detection of FPV, FHV, FCV, CDV, and CPV, with the protocol as follows: Place the test strips horizontally on a clean, dry, and flat surface, aspirate the sample supernatant using a pipette, and vertically add 4 drops of the mixture into the sample hole. Incubate for 10 min, and then interpret the results according to the following criteria: positive if both the control line (C) and test line (T) show coloration; negative if only C line shows coloration; invalid (retest required) if C line fails to develop color.

### 2.6. Fluorescent Immunoassay

Add the virus sample dropwise to the sample hole on the test strip. Insert the strip into the Getein 1600 VET Immunofluorescence Quantitative Analyzer (GeteinBiotech, Nanjing, China), and wait for the reaction to complete for automatic report generation. For whole blood samples of TOXO, *Leptospirosis*, *Canine babesiosis*, *Leishmania* spp., dilute with sample dilution buffer prior to adding to the test card sample hole, followed by detection using either the K9 Multi-channel Fluorescence Immunoassay Analyzer (Healvet, Guangzhou, China) or the FiDX Fluorescent Immunoassay Analyzer (GlinX, Shanghai, China).

### 2.7. Nucleic Acid Test

*Leishmania* spp. was detected using the *Leishmania* spp. Nucleic Acid Test Card (Pluslife, Guangzhou, China). The specific protocol was as follows: Add the sample to the Nucleic Acid Releasing Agent and mix thoroughly, heat the mixture at 85 °C for 10 min. The mixture was then added to the Reaction Card and incubated according to the manufacturer’s instructions. Finally, the Reaction Card was inserted into the Pluslife MiniDock instrument (Pluslife, Guangzhou, China) for detection and report generation.

### 2.8. Statistical Analysis

Data were analyzed via SPSS 30.0 (IBM Corp., Armonk, NY, USA). Chi-square tests evaluated associations between infection rates and age, sex, season, and vaccination status (*p* < 0.05 for inclusion in logistic regression). The Hosmer-Lemeshow test assessed model fit (*p* > 0.05 indicating adequacy). Odds ratios (OR) and 95% confidence intervals (CI) were calculated *(p* < 0.05 considered significant).

## 3. Results

### 3.1. Disease Proportions

**Figure 1:**
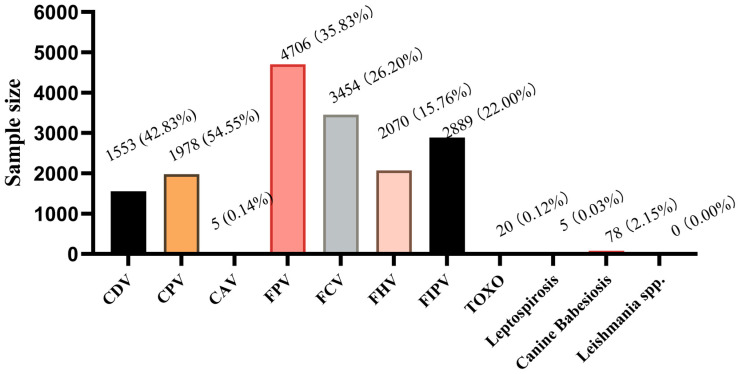
Disease Proportions.

FPV (35.83%, 4706/13,134) had the highest prevalence, followed by FCV (26.20%, 3454/13,134), FIPV (22.00%, 2889/13,134), and FHV (15.76%, 2070/13,134). In dogs, CPV and CDV were the most prevalent, with positivity rates of 54.55% (1978/3626) and 42.83% (1553/3626), respectively. Other infectious diseases, such as Toxoplasmosis (TOXO), accounted for 0.12% (20/16,760), while CAV-1, leptospirosis, and Canine Cabesiosis exhibited lower prevalence, and leishmaniasis tested negative (Figure 1).

### 3.2. Analysis of Common Feline Diseases

**Figure 2:**
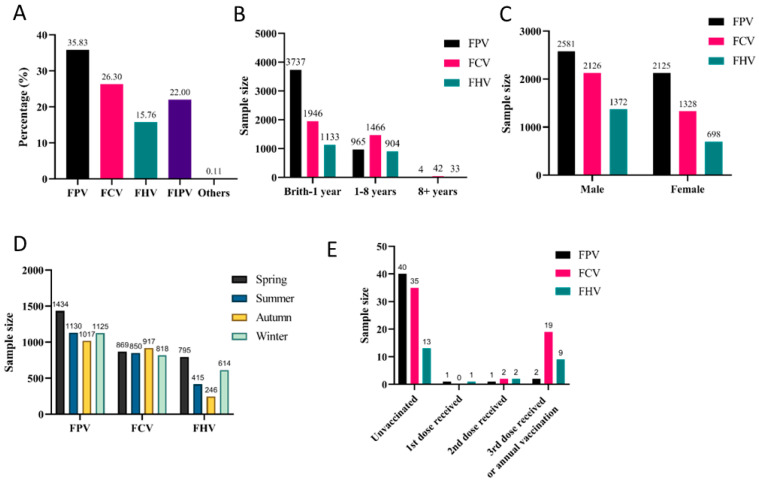
Prevalence of major feline viral infections: (**A**) Overall detection rates. (**B**) Age distribution. (**C**) Sex-associated differences. (**D**) Seasonal distribution. (**E**) Vaccination status.

Given that FPV, FCV, and FHV are the three target pathogens covered by the Fel-O-Vax^®^ PCT vaccine, their infection data were collectively presented in Section 3.2, with subsequent analysis of key influencing factors. Specifically, the majority of affected cats were under one year of age, with FPV, FCV, and FHV accounting for 79.41%, 56.34%, and 54.73% of cases, respectively (Figure 2B).

In this study, the incidence rates among male cats for FPV, FCV, and FHV were 54.84% (2581/4706), 61.55% (2126/3454), and 66.28% (1372/2070), respectively. Given that male cats accounted for 61.42% (8067/13,134) of the total sample population, we conclude that FCV infection rates show no significant sex association. However, male cats showed higher positivity rates for FHV, while female cats had a higher rate for FPV (Figure 2C).

Due to regional variations in seasonal temperatures, the reported seasonal prevalence patterns of the three major viral diseases differ. FPV, FCV, and FHV infections occurred throughout the year. FCV showed minimal seasonal variation in incidence rates, while FPV exhibited its highest positivity rate in spring (30.47%, 1434/4706). Coincidentally, FHV infections predominated during colder seasons, with peaks in spring (38.41%, 795/2070) and winter (29.66%, 614/2070) (Figure 2D).

In this study, analysis of cases with known vaccination status (Figure 2E) revealed that unvaccinated cats were more susceptible to all three viral diseases compared to vaccinated cats, despite the limited sample size of vaccination-confirmed cases. Remarkably, FCV and FHV showed higher infection rates among cats that had received three vaccine doses or annual boosters compared to FPV in the same vaccinated population.

**Figure 3:**
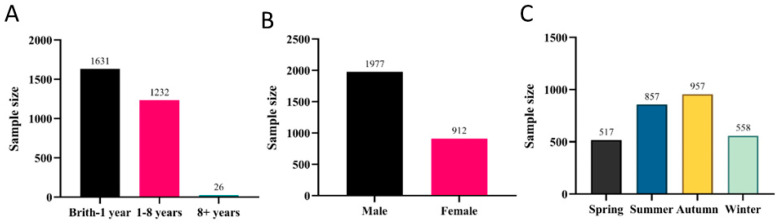
Prevalence of FIPV: (**A**) Age distribution. (**B**) Sex-associated differences. (**C**) Seasonal distribution.

Notably, FIPV demonstrated the third-highest infection rate at 22.00% (2889/13,134). Analysis of the collected data revealed that the disease mainly affected cats under one year of age (Figure 3A). Male cats exhibited significantly higher FIPV positivity rates (68.54%, 1980/2889) (Figure 3B). In Shenzhen, the highest incidence of FIPV infections in cats was observed during summer and autumn (Figure 3C), accounting for 29.66% (857/2889) and 33.13% (957/2889) of cases, respectively.

To further analyze the associations between FPV, FCV, FHV, and FIPV infections with the aforementioned factors, we initially performed univariate analysis using chi-square tests (Table 3), followed by logistic regression of infection-related variables (Table 4). For FPV, the infection risk in female cats was 1.484-fold higher than that in male cats (*p* < 0.001). Infection risk exhibited a significant decreasing trend with advancing age, as 1–8-year-old and ≥8-year-old cats demonstrated 0.332-fold and 0.048-fold the risk of cats <1 year old, respectively (*p* < 0.001). Additionally, the infection risk during summer, autumn, and winter was significantly lower than that in spring (0.748-fold, 0.658-fold, and 0.843-fold, respectively; *p* < 0.05). For FCV, infection risk increased significantly with age, with 1–8-year-old and ≥8-year-old cats having 1.599-fold and 2.268-fold higher risk compared to cats <1 year old (*p* < 0.001). Specifically, the infection risk in autumn was 1.361-fold higher than that in spring (*p* < 0.001), while no statistically significant association was observed between sex and FCV infection. For FHV, female cats had a 0.782-fold lower infection risk than male cats (*p* < 0.001). Infection risk rose with age, as 1–8-year-old and ≥8-year-old cats showed 1.493-fold and 2.765-fold higher risk relative to cats <1 year old (*p* < 0.001). Furthermore, the infection risk in summer and autumn was significantly lower than that in spring (0.529-fold and 0.308-fold; *p* < 0.001). For FIPV, the infection risk in female cats was 0.693-fold lower than that in male cats (*p* < 0.001). 1–8-year-old cats exhibited a 1.624-fold higher infection risk compared to cats <1 year old (*p* < 0.001). Notably, the infection risk during summer, autumn, and winter was significantly higher than that in spring (2.22-fold, 2.757-fold, and 1.311-fold, respectively; *p* < 0.001). The results demonstrated statistically significant associations between sex, age, and season with FPV, FHV, and FIPV infections. Similarly, age and season showed significant correlations with FCV infection, while no statistically significant association was observed between sex and FCV infection.

### 3.3. Analysis of Common Canine Diseases

**Figure 4:**
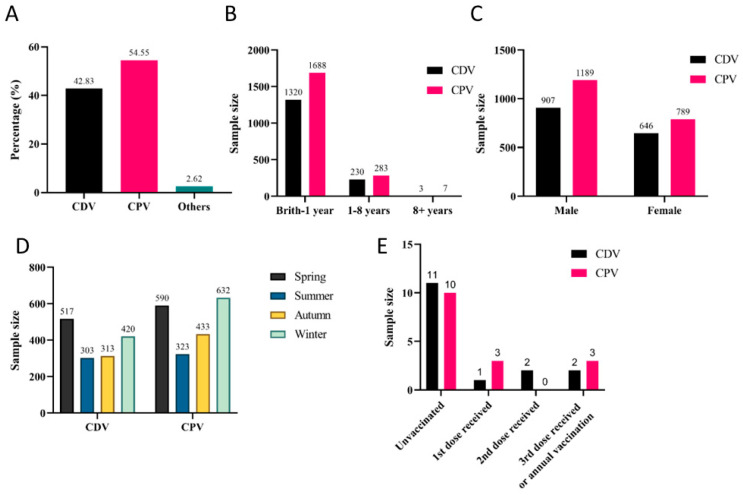
Prevalence of major canine viral infections: (**A**) Overall detection rates. (**B**) Age distribution. (**C**) Sex-associated differences. (**D**) Seasonal distribution. (**E**) Vaccination status.

Among the 3626 dog cases, CPV and CDV were the most prevalent, accounting for 54.55% (1553/3626) and 42.83% (1978/3626), respectively (Figure 4A). Both CDV and CPV exhibited the highest incidence rates in dogs under one year old, with positivity rates reaching 85% (1320/1553) and 85.34% (1688/1978), respectively (Figure 4B). Sex did not significantly influence CPV or CDV infections (Figure 4C). CDV infections showed no clear seasonal pattern, though the highest positivity rate (33.29%, 517/1553) was observed in spring. In contrast, CPV infections exhibited distinct seasonal peaks during winter (31.90%, 631/1978) and spring (29.78%, 589/1978) (Figure 4D). It should be noted that vaccination significantly reduced infection rates, though some vaccinated dogs still contracted the disease (Figure 4E).

The univariate analysis using chi-square tests for the aforementioned influencing factors revealed that age and season were significantly associated with CPV and CDV infections, while sex was not (Table 5). These variables were subsequently analyzed using logistic regression (Table 6). The data confirmed that dogs aged 1–8 years and those over 8 years had 0.801-fold (*p* = 0.017) and 0.240-fold (*p* = 0.001) risks of CPV infection, respectively, compared to dogs under 1 year old. For CDV infection, the risks were 0.887-fold (*p* = 0.204) and 0.148-fold (*p* = 0.002) for the same age groups relative to the under-1-year reference group. Compared to spring, winter showed a 1.336-fold higher risk for CPV infection (*p* < 0.001), while autumn and winter demonstrated 0.796-fold (*p* = 0.016) and 0.773-fold (*p* = 0.003) risks for CDV infection, respectively.

## 4. Discussion

Companion animals like dogs and cats play a vital role in modern society, providing emotional companionship and enhancing human social connections. However, their health remains persistently threatened by various infectious diseases, which not only compromise animal welfare and health but also pose potential risks to public health.

In this study, cats under one year of age demonstrated significantly higher risks of FPV, FCV, and FHV infections. And the infection rates decrease progressively with advancing age, a pattern consistent with prior research [18,19,20]. This increased susceptibility stems from immunological immaturity, loss of maternal antibody protection post-weaning, and incomplete vaccination, leading to an immunity gap. Furthermore, as this age period typically coincides with kitten adoption and household introduction, environmental stressors during the transition may further compromise immune function, increasing viral susceptibility [21]. Notably, FHV-infected cats exhibit particularly high recurrence rates when exposed to stress [22].

This paradox primarily stems from the failure of hospital-based case samples to fully cover high-risk kitten populations such as catteries, cat shelters, and strays [23]; the reduced clinical attendance of infected kittens due to high mortality, whereas adult cats are more likely to be detected by owners and presented to veterinary clinics due to chronic clinical manifestations, including FCV-associated stomatitis and FHV-induced corneal ulcers [24]; and the misclassification of stress-induced FHV-1 reactivation cases in adult cats as incident infections without distinguishing primary infections [25]. Notably, this finding constitutes a limitation of the present study, as these factors collectively distorted the age-effect estimates. While this does not contradict the theoretical premise that kittens have higher susceptibility, it underscores how human factors and selection biases can influence epidemiological data.

FCV showed no sex-based prevalence difference. Male cats are at higher risk of FHV infection due to their increased fighting behavior in multi-cat environments (leading to virus transmission through bites and scratches) [22,26] and greater outdoor exposure to pathogens [27]. While previous studies found no significant association between FPV infection and sex [18], our study observed a higher prevalence in female cats, interestingly. The risk may come from maternal behaviors such as grooming kittens or cleaning contaminated environments (fecal-oral transmission). Pregnant cats can transmit the virus vertically. Although protected by maternal antibodies, infected kittens may remain viral carriers for up to two months postpartum [2].

Seasonal analysis revealed relatively stable FCV incidence across seasons, while FPV showed peak prevalence in spring (30.47%), consistent with Li et al.’s findings [28]. As a parvovirus, FPV can survive in the environment for over a year [2]. Spring in Shenzhen features high humidity (95–100% RH), creating ideal conditions for viral persistence in cat litter and owners’ shoe soles, facilitating indirect transmission to indoor cats. While Cao et al. reported no seasonal variation in FHV prevalence [29], our study identified peak infections during cold seasons (spring: 38.41%; winter: 29.66%). For one thing, compromised mucosal immunity is due to thermoregulatory stress from large diurnal temperature variations in spring. For another, as a highly stable DNA virus, FHV can maintain persistent shedding even during latent infection. In multi-cat households, virus-laden aerosols generated by infected cats through coughing or sneezing may remain suspended for hours in poorly ventilated indoor environments during winter, establishing potential transmission chains [30,31].

In China, the Fel-O-Vax^®^ PCT serves as the primary preventive measure against FPV, FCV, and FHV, and remains the only approved imported feline vaccine in the country. Our results confirm that vaccination significantly reduces infection rates for all three viruses. However, breakthrough infections still occur, particularly with FCV and FHV—a phenomenon also documented in surveillance studies by Li and Wang [28,32], suggesting potential antigenic divergence between circulating field strains and vaccine strains. Recent studies have reported cases of FCV and FHV infections in cats despite inactivated vaccination [22]. This vaccine failure may be attributed to FCV’s nature as an RNA virus, which exhibits high mutation rates that frequently generate immune-escaping variants, thereby limiting vaccine effectiveness [28,33]. Following FHV infection in cats, while antibodies are generated, the virus establishes lifelong latency primarily within the trigeminal and vestibular ganglia [34,35]. This persistent infection can reactivate during periods of stress or immunosuppression, leading to recurrent clinical disease [36]. Additional studies indicate that while vaccines can mitigate clinical symptoms, they fail to provide complete protection, and vaccine-induced immunity may wane over time [37]. Based on these findings, we suggest booster vaccination every three years for low-risk cats (indoor/solitary) and more frequently for high-risk cats (multi-cat households/shelters) [38]. More importantly, enhanced hygiene, isolation protocols, and environmental disinfection should complement vaccination for optimal prevention [39].

FIPV infection ranked third in prevalence (22.00%) in our study. Both our data and Rohrbach et al.’s report [40] identified the highest disease incidence in young cats under 1 year, with significantly lower rates in cats over 8 years. Male cats showed markedly higher FIPV positivity than females, corroborating previous epidemiological reports [40,41]. Male cats exhibit weaker immune responses to FIPV, resulting in impaired viral clearance that leads to persistent infection and consequently higher seropositivity rates. Seasonal analysis revealed that compared to spring, the risk of FIPV infection was 2.22-fold higher in summer and 2.757-fold higher in autumn, potentially attributable to Shenzhen’s warm-humid climate favoring viral survival and increased feline activity facilitating transmission. While FIPV research remains limited, its pathogenesis undoubtedly involves complex virus–host-environment interactions [42].

In dogs, CPV and CDV were most prevalent in puppies (<1 year old), consistent with Cavalli et al.’s findings [43]. This age-related susceptibility is primarily attributed to the immature immune function of puppies [44] and incomplete protection from maternal antibodies [45]. CDV infections showed no clear seasonal pattern but peaked in spring, while CPV exhibited higher prevalence in winter and spring. This seasonality can be attributed to cold resistance of both viruses [46] and potential immunosuppression during dogs’ spring breeding season. Additionally, warmer spring weather increases canine outdoor activity, elevating exposure risks to virus-contaminated fomites [47].

Our study found no significant association between CDV/CPV infections and gender in dogs, a finding consistent with a serosurvey conducted by Kim et al. on military dogs in South Korea [48]. However, a study by Ki Oleiwi et al. [49] found that the infection rate of CPV in male dogs (65.36%) was significantly higher than that in female dogs. They attributed this difference to male dogs’ roaming and territorial behaviors, as well as the preference for rearing male dogs, which collectively increase the risk of CPV exposure in canines.

Vaccination is a key preventive measure for canine viral diseases. Currently, various commercial vaccines are widely used in dogs. The most common ones in China are canine combination vaccines Vanguard^®^ 5/8, which protect against multiple pathogens such as CDV, CPV, canine adenovirus (CAV), and canine parainfluenza virus (CPIV). However, the protective efficacy of vaccines is influenced by multiple factors, and two critical factors may lead to immunization failure: (1) the high mutability of RNA viruses (CDV) and single-stranded DNA viruses (CPV), potentially causing antigenic mismatch between vaccine strains and local circulating variants [50]; and (2) interference from maternal antibodies [51].

Vaccination remains the most effective strategy for preventing and controlling common canine and feline pathogens. The World Small Animal Veterinary Association (WSAVA) designates vaccines against CDV, CPV, CAV-1, FPV, FCV, and FHV as core vaccines, essential for all pets regardless of environmental or geographical factors [52]. Based on these findings, we recommend: ensuring timely vaccination of young pets with regular boosters adjusted for lifestyle factors such as indoor/outdoor living and single/multi-pet households; enhancing public education on disease prevention to integrate improved hygiene protocols, isolation of infected animals, and environmental disinfection with vaccination programs; and maintaining particular vigilance during winter and spring when infectious diseases peak, as temperature fluctuations may compromise immunity and increase viral susceptibility.

In the present study, the overall infection rate of parasitic zoonoses was relatively low, ranging from 0% to 2.15%. Canine babesiosis and toxoplasmosis were the predominant infections, while no Leishmania-positive cases were detected. Canine babesiosis is a significant tick-borne disease caused by various species of the protozoan genus *Babesia* [53]. Canine babesiosis is widely prevalent primarily in Asia and Europe, with an infection rate ranging from 2% to 60% [54]. Toxoplasmosis, caused by *Toxoplasma gondii*, is one of the most prevalent parasitic infections in humans worldwide [55]. As the definitive host of T. gondii, cats can excrete oocysts in their feces, which contributes to environmental contamination and thereby increases the risk of human infection [56]. Su et al.’s survey report indicates that, in mainland China, the overall T. gondii infection prevalence in pet dogs is 0.56% to 27.65%, while that in pet cats is 2.50% to 60.00% [57]. Visceral leishmaniasis (VL), caused by *Leishmania* spp., is an important vector-borne zoonotic disease. The domestic dog is the main reservoir for human infection [58]. Through the implementation of a series of rigorous and effective control measures, the Chinese government has essentially eliminated Human VL from most endemic areas, with only sporadic cases remaining in the mountainous regions of western China [59]. According to official records in 2023, the national annual incidence rate of the disease is extremely low, at merely 0.0196/100,000 population (277 cases in total) [60].The low prevalence of parasitic zoonoses not only reflects the relatively low risk of parasitic vector exposure in urban domestic pets but also indicates that the potential risk of cross-species transmission of such zoonotic diseases still requires attention, particularly in dog-cat cohabitation scenarios.

There are some limitations in this study. Firstly, Insufficient data standardization: Medical record standards and symptom description details vary across clinical institutions, limiting the collection of key variables in the sample database. Additionally, clinical symptoms of infected animals exhibit high heterogeneity due to individual differences and infection stages, hindering standardized extraction and thus were not included in the statistical analysis of this study. Secondly, biases exist in sample representativeness and diagnostic accuracy: Clinical samples did not cover high-risk groups such as shelters and strays; adult cats had a higher consultation rate, and FHV-1 reactivation cases may be misclassified as primary infections, potentially distorting the estimation of age effects. These limitations have compromised the completeness and reliability of the study results. In subsequent research, we will collaborate with clinical institutions to establish unified medical record standards, expand and refine sample sources, and enhance the collection of risk factor-related data, thereby comprehensively revealing key influencing factors of canine and feline infectious disease prevalence and further enhancing the scientific rigor and practical value of the research.

## 5. Conclusions

In summary, this study investigated the prevalence of 11 common pathogens in dogs and cats. Risk factor analysis revealed that sex, age, and season were significantly associated with FPV, FHV, and FIPV infections, while age and season were linked to FCV, CPV, and CDV infections (sex showed no association). Notably, we found that female cats exhibited a distinct susceptibility to FPV. Seasonal analysis indicated a higher prevalence of FHV in winter and spring, whereas FIPV peaked in summer and autumn. These findings differ from previous studies and provide new insights into the epidemiological patterns of companion animal infectious diseases, particularly regarding population-specific susceptibility and seasonal transmission dynamics. The results hold significant implications for developing targeted prevention and control strategies.

## Figures and Tables

**Table 1:** The distribution of case samples.

Variety	Category	Dog (*n* = 3626)		Cat (*n* = 13,134)	
		Proportion	Frequency (%)	Proportion	Frequency (%)
Sex	Male	2154	59.40	8067	61.42
	Female	1472	40.60	5067	38.58
Age	<1 year	3033	83.65	8450	64.34
	1–8 years	563	15.53	4579	34.86
	≥8 years	30	0.83	105	0.80
Season	Spring	1128	31.11	3618	27.55
	Summer	654	18.04	3253	24.77
	Autumn	781	21.54	3142	23.92
	Winter	1063	29.32	3121	23.76

**Table 2:** The vaccination status of case samples.

Category	Dog (*n* = 32)		Cat (*n* = 141)	
	Proportion	Frequency (%)	Proportion	Frequency (%)
Unvaccinated	21	65.63	100	70.92
1st dose received	4	12.50	3	2.13
2nd dose received	2	6.25	5	3.55
3rd dose received or annual vaccination	5	15.63	33	23.40

**Table 3:** Chi-square Analysis of Factors Associated with Feline Infectious Diseases.

Disease	Variety	Category	Proportion	Frequency (%)	*χ* ^2^	*p*-Value
FPV			4706/13,134	35.83		
	Sex	Male	2581/8067	31.99	133.832	<0.001
		Female	2125/5067	41.94		
	Age	<1 year	3737/8450	44.22	739.432	<0.001
		1–8 years	965/4579	21.07		
		≥8 years	4/105	3.81		
	Season	Spring	1434/3618	39.64	40.917	<0.001
		Summer	1130/3253	34.74		
		Autumn	1017/3142	32.37		
		Winter	1125/3121	36.05		
FCV			3454/13,134	26.30		
	Sex	Male	2126/8067	26.35	0.034	0.854
		Female	1328/5067	26.21		
	Age	<1 year	1946/8450	23.03	133.978	<0.001
		1–8 years	1466/4579	32.02		
		≥8 years	42/105	40.00		
	Season	Spring	869/3618	24.02	23.27	<0.001
		Summer	850/3253	26.13		
		Autumn	917/3142	29.19		
		Winter	818/3121	26.21		
FHV			2070/13,134	15.76		
	Sex	Male	1372/8067	17.01	24.488	<0.001
		Female	698/5067	13.78		
	Age	<1 year	1133/8450	13.41	109.311	<0.001
		1–8 years	904/4579	19.74		
		≥8 years	33/105	31.43		
	Season	Spring	795/3618	21.97	312.137	<0.001
		Summer	415/3253	12.76		
		Autumn	246/3142	7.83		
		Winter	614/3121	19.67		
FIPV			2889/13,134	22.00		
	Sex	Male	1977/8067	24.51	76.834	<0.001
		Female	912/5067	18.00		
	Age	<1 year	1631/8450	19.30	100.54	<0.001
		1–8 years	1232/4579	26.91		
		≥8 years	26/105	24.76		
	Season	Spring	517/3618	14.29	323.053	<0.001
		Summer	857/3253	26.34		
		Autumn	957/3142	30.46		
		Winter	558/3121	17.88		

Variables with *p* < 0.05 were considered for inclusion in the logistic regression analysis.

**Table 4:** Logistic Regression Analysis of Risk Factors for Feline Infectious Diseases.

Disease	Variety	Category	*p*-Value	OR	95%CI
FPV	Sex	Male	Ref		
		Female	<0.001	1.484	1.376, 1.599
	Age	<1 year	Ref		
		1–8 years	<0.001	0.332	0.305, 0.361
		≥8 years	<0.001	0.048	0.018, 0.13
	Season	Spring	Ref		
		Summer	<0.001	0.748	0.676, 0.829
		Autumn	<0.001	0.658	0.593, 0.73
		Winter	0.001	0.843	0.761, 0.935
FCV	Sex	Male			
		Female			
	Age	<1 year	Ref		
		1–8 years	<0.001	1.599	1.475, 1.733
		≥8 years	<0.001	2.268	1.529, 3.366
	Season	Spring	Ref		
		Summer	0.01	1.156	1.035, 1.29
		Autumn	<0.001	1.361	1.22, 1.518
		Winter	0.031	1.13	1.011, 1.263
FHV	Sex	Male	Ref		
		Female	<0.001	0.782	0.707, 0.864
	Age	<1 year	Ref		
		1–8 years	<0.001	1.493	1.354,1.646
		≥8 years	<0.001	2.765	1.807,4.232
	Season	Spring	Ref		
		Summer	<0.001	0.529	0.464, 0.602
		Autumn	<0.001	0.308	0.264, 0.359
		Winter	0.02	0.868	0.771, 0.978
FIPV	Sex	Male	Ref		
		Female	<0.001	0.693	0.634, 0.758
	Age	<1 year	Ref		
		1–8 years	<0.001	1.624	1.489, 1.771
		≥8 years	0.069	1.524	0.967, 2.402
	Season	Spring	Ref		
		Summer	<0.001	2.22	1.964, 2.51
		Autumn	<0.001	2.757	2.442, 3.114
		Winter	<0.001	1.311	1.149, 1.495

*p* < 0.05 was considered significant. OR, odds ratio; CI, confidence interval.

**Table 5:** Chi-square Analysis of Factors Associated with Canine Infectious Diseases.

Disease	Variety	Category	Proportion	Frequency (%)	*χ* ^2^	*p*-Value
CPV			1978/3626	54.55		
	Sex	Male	1189/2154	55.20	0.902	0.342
		Female	789/1472	53.60		
	Age	<1 year	1688/3033	55.65	17.450	<0.001
		1–8 years	283/563	50.27		
		≥8 years	7/30	23.33		
	Season	Spring	590/1128	52.30	19.884	<0.001
		Summer	323/654	49.39		
		Autumn	433/781	55.44		
		Winter	632/1063	59.45		
CDV			1553/3626	42.83		
	Sex	Male	907/2154	42.11	1.129	0.288
		Female	646/1472	43.89		
	Age	<1 year	1320/3033	43.52	14.696	<0.001
		1–8 years	230/563	40.85		
		≥8 years	3/30	10.00		
	Season	Spring	517/1128	45.83	14.628	0.002
		Summer	303/654	46.33		
		Autumn	313/781	40.08		
		Winter	420/1063	39.51		

Variables with *p* < 0.05 were considered for inclusion in the logistic regression analysis.

**Table 6:** Logistic Regression Analysis of Risk Factors for Canine Infectious Diseases.

Disease	Variety	Category	*p*-Value	OR	95%CI
CPV	Sex	Male			
		Female			
	Age	<1 year	Ref		
		1–8 years	0.017	0.801	0.668, 0.96
		≥8 years	0.001	0.240	0.102, 0.562
	Season	Spring	Ref		
		Summer	0.206	0.882	0.727, 1.071
		Autumn	0.184	1.133	0.942, 1.363
		Winter	<0.001	1.336	1.128, 1.583
CDV	Sex	Male			
		Female			
	Age	<1 year	Ref		
		1–8 years	0.204	0.887	0.738, 1.067
		≥8 years	0.002	0.148	0.045, 0.49
	Season	Spring	Ref		
		Summer	0.825	1.022	0.842, 1.241
		Autumn	0.016	0.796	0.661, 0.959
		Winter	0.003	0.773	0.652, 0.916

*p* < 0.05 was considered significant. OR, odds ratio; CI, confidence interval.

## Data Availability

The data presented in this study are available from the corresponding authors on request.
